# The role of ubiquitin and the 26S proteasome in plant abiotic stress signaling

**DOI:** 10.3389/fpls.2014.00135

**Published:** 2014-04-16

**Authors:** Sophia L. Stone

**Affiliations:** Department of Biology, Dalhousie UniversityHalifax, NS, Canada

**Keywords:** abiotic stress, abscisic acid, E3 ubiquitin ligase, 26S proteasome, protein degradation, ubiquitination

## Abstract

Ubiquitin is a small, highly conserved, ubiquitously expressed eukaryotic protein with immensely important and diverse regulatory functions. A well-studied function of ubiquitin is its role in selective proteolysis by the ubiquitin-proteasome system (UPS). The UPS has emerged as an integral player in plant response and adaptation to environmental stresses such as drought, salinity, cold and nutrient deprivation. The UPS has also been shown to influence the production and signal transduction of stress-related hormones such as abscisic acid. Understanding UPS function has centered mainly on defining the role of E3 ubiquitin ligases, which are the substrate-recruiting component of the ubiquitination pathway. The recent identification of stress signaling/regulatory proteins that are the subject of ubiquitin-dependent degradation has increased our knowledge of how the UPS facilitates responses to adverse environmental conditions. A brief overview is provided on role of the UPS in modulating protein stability during abiotic stress signaling. E3 ubiquitin ligases for which stress-related substrate proteins have been identified are discussed.

## INTRODUCTION

The covalent attachment of ubiquitin molecules to selected proteins (referred to as ubiquitination) can influence activity, abundance, trafficking, or localization. The versatility of the ubiquitination pathway lies in the different ways in which ubiquitin molecules can be attached to a selected substrate protein (Komander and Rape, 2012). A single ubiquitin molecule can be attached to one (monoubiquitination) or multiple (multimonoubiquitination) lysine residues within a substrate protein. Another type of modification is the assembly of a chain of ubiquitin molecules (polyubiquitination) on a specific lysine residue within the substrate protein. Ubiquitin contains seven lysine residues each of which can be used to create ubiquitin-ubiquitin linkages, producing structurally diverse polyubiquitin chains (Nakasone et al., 2013). A polyubiquitin chain can be homogeneous using the same lysine residue to build the polymer, or of mixed topology with different lysine residues used to create ubiquitin-ubiquitin linkages. The significance of every type of modification is unknown. However, of the modifications that are understood, each confers a distinct outcome on a specific substrate protein. For example, monoubiquitination, or the attachment of a lysine 63-linked polyubiquitin chain, may serve as a signal for intracellular trafficking or protein activation, respectively (Chen and Sun, 2009). The assembly of a lysine 48-linked polyubiquitin chain is known to signal for the destruction of the modified protein (Thrower et al., 2000).

Ubiquitin-dependent protein degradation involves two distinct and successive steps: the attachment of a polyubiquitin chain consisting of at least four lysine 48-linked ubiquitin molecules to the substrate protein and degradation of the modified protein by the 26S proteasome, a large multi-catalytic protease complex. At the cellular level, the ubiquitin-proteasome system (UPS) is an essential part of regulatory networks that carefully controls the abundance of important enzymes, structural, and regulatory proteins. Plants utilize the UPS to facilitate changes in cellular protein content required for continuous growth, development, and adaptation to their ever changing environment (Stone and Callis, 2007; Vierstra, 2009). In the model research plant *Arabidopsis thaliana* (*At*; *Arabidopsis*), almost 6% of the genome is dedicated to the UPS (Hua and Vierstra, 2011). The majority of these genes encode for ubiquitin ligases (E3s), a central component of the ubiquitination pathway. Recently, E3s have emerged as modulators of plant response to abiotic stresses including drought, cold, salinity, heat, radiation, and nutrient deprivation (Yee and Goring, 2009; Lyzenga and Stone, 2012). Importantly, the action of a single E3 can regulate plant responses to multiple abiotic stresses. The impact of the UPS on abiotic stress tolerance is usually associated with regulating the actions of stress hormones such as abscisic acid (ABA). The significance of the UPS is further exemplified by the finding that multiple ubiquitin ligases are involved in regulating stress hormone signaling. Our understanding of how the UPS facilitate plant responses to various abiotic stresses is aided by recent studies that identified substrates for stress-related E3s. This review provides a brief overview of the role of these E3 ligase-substrates pairings during plant responses to abiotic stresses.

## THE UBIQUITIN ENZYMES

**FIGURE 1:**
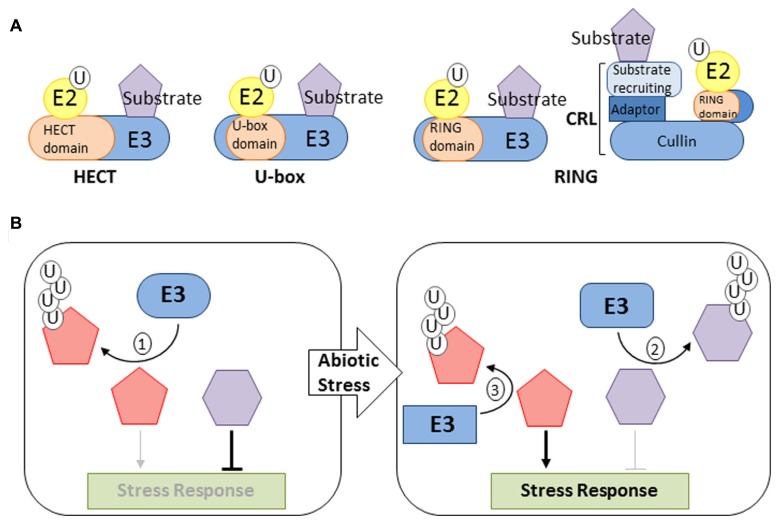
**Function of E3 ligases in abiotic stress response. (A)** Most common type of plant E3s. Ubiquitin ligases are categorized based on the presence of a RING, HECT, or U-box E2-binding domain. RING and U-box domain-containing E3s mediate transfer of ubiquitin (U) directly from the E2-Ub intermediate to the substrate protein. HECT domain-containing E3s form an E3-Ub intermediate prior to the transfer of ubiquitin to the substrate protein. RING domains are found in monomeric E3s and multisubunit CRLs. **(B)** Illustrations of the most common modes of action for E3 ligases in regulating abiotic stress responses. (1) E3 ligases may function as a negative response regulators required to supress stress response pathways by targeting positive regulators for degradation. (2) E3 ligases may promote stress signaling by functioning as positive response regulators that target negative regulators for degradation following stress perception. (3) E3 ligases may also function to attenuate stress signaling by targeting positive regulators for degradation.

Ubiquitination is a multi-step process involving the sequential action of three enzymes: E1 (ubiquitin activating enzyme; UBA), E2 (ubiquitin conjugating enzyme; UBC), and E3 (ubiquitin ligase). The conjugation process begins with the activation of ubiquitin by the E1 followed by transfer of ubiquitin to the E2, forming a thioester linked E2-ubiquitin (E2-Ub) intermediate. The substrate-recruiting E3 interacts with the E2-Ub allowing for the transfer of ubiquitin to the target (**Figure 1A**). Following the attachment of the initial ubiquitin molecule, the process can be repeated to assemble a polyubiquitin chain (Komander and Rape, 2012). The conjugation process is also reversible. Proteases referred to as deubiquitinating enzymes (DUBs) are able to cleave ubiquitin molecules from modified proteins (Reyes-Turcu et al., 2009). The ubiquitination pathway is hierarchical in that eukaryotic genomes are found to contain one or two E1, 10s of E2 and 100s of E3 encoding genes. For example, the *Arabidopsis* genome is predicted to encode for two E1 isoforms, 37 E2 enzymes and over 1300 E3s or components of E3 complexes (Hatfield et al., 1997; Kraft et al., 2005; Stone et al., 2005; Hua and Vierstra, 2011). The large number of ubiquitin enzymes suggests that many cellular processes are regulated via protein ubiquitination.

The capacity of the ubiquitination pathway to differentially modify numerous proteins is made possible by the abundance and diversity of ubiquitin ligases. The majority of plant E3s are of the homology to E6-associated carboxy-terminus (HECT), U-box, or Really Interesting New Gene (RING) type (**Figure 1A**). The *Arabidopsis* genome is predicted to encode for 7 HECT-type and 64 U-box-type E3s (Downes et al., 2003; Mudgil et al., 2004). Over 470 *Arabidopsis* genes are predicted to encode for RING domain-containing proteins (Stone et al., 2005). Ubiquitin ligases that utilize a RING domain for E2 binding can occur as monomeric E3s or multi-subunit Cullin (CUL) based RING E3 ligases (CRLs; **Figure 1A**). Three types of CRLs have been described in plants, each utilizing a different CUL subunit, CUL1, CUL3a/b, or CUL4 (Hotton and Callis, 2008; Hua and Vierstra, 2011). Each CUL serves as a platform upon which the RING domain-containing (RBX1a/b) and substrate-recruiting sub-units assemble (**Figure 1A**). Substrate-recruiting proteins utilized by plant CRLs belong to either the F-box, Broad complex Tramtrack Bric-a-Brac (BTB), or DDB1 binding WD40 (DWD) families. The F-box family is the largest with over 700 members followed by the DWD and BTB with 85 and 80 members, respectively (Lechner et al., 2006; Gingerich et al., 2007; Lee et al., 2008). The CUL1 based E3s (also referred to as Skp1-Cullin-F-box [SCF]) use the adaptor protein *Arabidopsis* S-Phase kinase-associated protein (ASK) to bind to F-box proteins (Bai et al., 1996; Lechner et al., 2006). CUL4 based E3s are assembled using DNA-damage binding (DDB1) as an adaptor to bind DWD proteins, while CUL3a/b interacts directly with BTB proteins (Gingerich et al., 2007; Lee et al., 2008). The large number of substrate-recruiting subunits and the ability to assemble E3 complexes using one of three CUL proteins makes the CRL group the largest class of ubiquitin ligases.

## THE UBIQUITINATION PATHWAY AND ABIOTIC STRESS TOLERANCE

A plants ability to survive abiotic stresses such as salinity, radiation, heavy metals, nutrient deprivation, cold, and drought relies heavily on proteomic plasticity. The UPS plays a crucial role in enabling plants to alter their proteome in order to effectively and efficiently perceive and respond to environmental stresses (Smalle et al., 2003; Kurepa et al., 2008). How the UPS functions to facilitate responses to a particular stress depends upon the nature of the substrate protein. For example, ubiquitin-dependent degradation of a positive regulator may serve to supress the response pathway until a stress stimulus is perceived (**Figure 1B**). In this case, ubiquitination of the substrate would cease allowing for accumulation of the regulatory protein and promotion of cellular changes required to acclimate the plant to external conditions. The ubiquitin ligase involved in modifying the regulatory protein would be designated a negative response regulator (Chen and Hellmann, 2013). On the other hand, ubiquitin ligase targeting a negative regulator for degradation in response to a stimulus would enable the activation of signaling pathways required for tolerance of the perceived stress (**Figure 1B**). Many examples of the aforementioned scenario have been reported, some of which are discussed below. Instances of the UPS functioning to attenuate stress signaling have also been described. In these cases, ubiquitin-dependent degradation of a positive regulator occurs following perception of a stress stimulus (**Figure 1B**). Maintenance of a certain level of signal intensity and termination of signal transduction would enable plants to recover and resume normal growth and development once environmental conditions improve.

One of the first indications of the importance of the ubiquitination pathway to abiotic stress tolerance is the finding that *ubiquitin* gene expression is up-regulated in plants exposed to high temperature stress (Genschik et al., 1992; Sun and Callis, 1997). In fact, overexpression of ubiquitin has been shown to increase plant tolerance of salinity and drought conditions (Guo et al., 2008). Since this finding, stress-related roles have been demonstrated for a number of ubiquitin enzymes. Many E2 encoding genes are stress-inducible. Transcript levels of *Glycine max UBC2* (*GmUBC2*; soybean), *Arachis hypogaea UBC2* (*AhUBC2;* peanut) and *Arabidopsis UBC32* (*AtUBC32*) are up-regulated in response to drought and/or salt stress (Zhou et al., 2010; Wan et al., 2011; Cui et al., 2012). Overexpression of *AtUBC32* rendered plants sensitive to salt stress (Cui et al., 2012). Conversely, *AtUBC32* mutant plants are more tolerant of salt stress. Also, transgenic *Arabidopsis* plants overexpressing *Vigna radiata UBC1* (*VrUBC1*; mung bean)*, AhUBC2,* or *GmUBC2* were more tolerant of drought stress (Zhou et al., 2010; Wan et al., 2011; Chung et al., 2013). An increasing number of ubiquitin ligases have been shown to be involved in plant responses to various abiotic stresses. A number of excellent review articles provide a detailed listing of many of these E3s (Yee and Goring, 2009; Lee and Kim, 2011; Lyzenga and Stone, 2012; Chen and Hellmann, 2013). This review focuses on examples of E3 ligases for which stress-related substrate proteins have been identified.

Plant response to adverse environmental conditions is a complex and coordinated process involving activation of signaling networks and changes in the expression of hundreds of genes. By modulating the abundance of transcription factors, the UPS may affect the changes in gene expression required to mitigate the potential negative effects of environmental stress. E3 ligases may prohibit transcription activity by targeting the transcription factor for degradation under non-stress conditions. A well-described example is the regulation of dehydration-responsive element binding protein (DREB) 2A by the RING-type E3 ligases DREB2A-interacting protein (DRIP) 1 and DRIP2 (Qin et al., 2008; Morimoto et al., 2013). DREB2A is a transcription factor that regulates the expression of many drought and salt stress-inducible genes (Sakuma et al., 2006a,b). In accordance with UPS regulation, DREB2A only accumulates in transgenic plants treated with proteasome inhibitors (Sakuma et al., 2006a,b; Qin et al., 2008). DRIP1 and DRIP2 are capable of attaching ubiquitin molecules to DREB2A in *in vitro* ubiquitination assays (Qin et al., 2008). Furthermore, DREB2A is stable in *drip1drip2* plants and drought tolerance of the double mutant is further enhanced by overexpression of the transcription factor (Qin et al., 2008). This demonstrates that DREB2A is unstable under non-stress conditions and DRIP1/2 targets the transcription factor for degradation. Exposure to abiotic stresses such as heat and drought stabilize DREB2A and levels of the transcription factor remain elevated during the stress period (Sakuma et al., 2006a; Morimoto et al., 2013). The mechanism underlying the stress-induced stabilization of DREB2A is not known. DRIP1 and DRIP2 localize to and interact with DREB2A within the nucleus (Qin et al., 2008). DREB2A lacking two nuclear localization signals (NLSs) is observed in the cytosol and is more stable compared to the wild type transcription factor (Morimoto et al., 2013). Therefore, under non-stress conditions, DREB2A degradation seems to occur mainly within the nucleus (Qin et al., 2008; Morimoto et al., 2013). A possible mechanism for DREB2A stabilization is stress-induced relocalization of the DRIP1 and DRIP2 to the cytosol. Alternatively, stress-induced ubiquitin-dependent degradation of DRIP1 and DRIP2 may occur within the nucleus. Another example is *Botrytis* Susceptible1 (BOS1), a nuclear-localized R2R3MYB transcription factor that is required for tolerance of drought, salt and oxidative stresses (Mengiste et al., 2003). To demonstrate proteasome-dependent turnover of BOS1, the stability of the transcription factor was assessed in planta using a β-glucuronidase (GUS) reporter system. GUS activity was only detected following treatment with proteasome inhibitors, which indicate inhibition of BOS1 degradation (Luo et al., 2010). Botrytis Susceptible1 Interactor (BOI) is a nuclear-localized RING-type E3 that interacts with BOS1 in plant cells (Luo et al., 2010). BOI is capable of attaching ubiquitin molecules to BOS1 in *in vitro* assays (Luo et al., 2010). Consistent with a role in regulating BOS1 abundance, reduction in *BOI1* expression resulted in reduced tolerance of salt stress (Luo et al., 2010). These results suggest that BOI1 mediate the ubiquitin-dependent turnover of BOS1 under non-stress conditions. Stress-induced stabilization of BOS1 has not been reported.

The UPS involvement in regulating responses to abiotic stresses extends beyond the proteolysis of transcription factors. The RING-type E3 ligases *Arabidopsis* Toxicos EN Levadura (ATL) 6 and ATL31 control the abundance of a 14-3-3 protein required for seedling response to carbon/nitrogen (C/N) stress (Sato et al., 2009, 2011; Maekawa et al., 2012). The ratio between carbon and nitrogen is tightly regulated and changes in availability disrupt early seedling establishment causing post-germinative growth arrest (Coruzzi and Bush, 2001). Overexpression of 14-3-3χ results in hypersensitivity to C/N stress (Sato et al., 2011). Accordingly, loss of *ATL6* and *ATL31* results in hypersensitivity to C/N stress and overexpression of the 14-3-3χ exaggerates the phenotypes of *atl6atl31* (Sato et al., 2011; Maekawa et al., 2012). Further evidence for ATL6/ATL31-mediated turnover of 14-3-3χ includes ubiquitination of 14-3-3χ by ATL6 and ATL31 during *in vitro* assays and accumulation of 14-3-3χ in *atl6atl31* seedlings (Sato et al., 2011).14-3-3χ protein levels increase in wild type seedlings exposed to C/N stress. Importantly, the C/N stress-induced increase in 14-3-3χ levels does not occur in *atl6atl31* seedlings. This suggests that ATL6/31 mediates the turnover of 14-3-3χ under non-stress conditions and degradation is prohibited during exposure to C/N stress. Another example is *Oryza sativa* drought-induced SINA protein 1 (OsDIS1), a RING-type E3 with high sequence similarity to *Arabidopsis* SINAT5 (Ning et al., 2011). Loss of *OsDIS1* function increased drought tolerance in rice plants. Conversely, transgenic rice plants overexpressing *OsDIS1* displayed reduced drought tolerance. A search for OsDIS1 interacting proteins identified OsNek6, a microtubule-associated serine/threonine protein kinase that belongs to the Never in Mitosis gene A-related kinase family (Vigneault et al., 2007). *Arabidopsis* Nek6 (AtNek6) was previously shown to be involved in microtubule-dependent morphogenesis of epidermal cells (Sakai et al., 2008). However, a positive role for AtNek6 in salt stress response has been reported (Lee et al., 2010; Ning et al., 2011). OsNek6 is degraded by the 26S proteasome and OsDIR1 does contribute to OsNex6 turnover in the absence of stress (Ning et al., 2011). A role for OsNex6 in plant response to drought stress was not reported, however OsDIS1-mediated turnover may function to suppress OsNex6 activity until stress conditions arise.

Ubiquitin-dependent degradation also functions to attenuate stress signaling. An example of this is the RING-type E3 ligase high expression of osmotically responsive gene 1 (HOS1), which mediates the degradation of Inducer of CBF Expression 1 (ICE1), a MYC transcription factor that regulates the expression of cold-responsive genes. HOS1 is capable of catalyzing ICE1 ubiquitination *in vitro* and *in vivo* (Dong et al., 2006). Consistent with a role in mediating ICE1 degradation, overexpression of HOS1 results in reduced expression of cold-responsive genes and increased sensitivity to freezing conditions (Dong et al., 2006). Exposure to cold stress up-regulates *ICE1* expression, however, low temperatures also promote proteasome-dependent degradation of the transcription factor (Chinnusamy et al., 2003; Dong et al., 2006). Turnover of nuclear-localized ICE1 is facilitated by cold-induced relocalization of HOS1 from the cytoplasm to the nucleus (Lee et al., 2001; Dong et al., 2006). The cold-induced HOS1-mediated degradation of ICE1 is suggested to facilitate the transient expression of cold-responsive genes (Chinnusamy et al., 2003; Dong et al., 2006). Another substrate for HOS1 is Constans (CO), a transcription factor that promotes flowering (Putterill et al., 1995; Jung et al., 2012; Lazaro et al., 2012). HOS1 interacts directly with and ubiquitinates CO (Jung et al., 2012; Lazaro et al., 2012). HOS1 regulation of CO abundance provides an explanation for the early flowering phenotype of *hos1* plants (Lee et al., 2001; Lazaro et al., 2012). Similar to the regulation of ICE1, exposure to low temperature promotes HOS1-dependent proteasomal degradation of CO (Jung et al., 2012). HOS1 regulation of CO abundance provides a link between cold stress response and control of flowering.

Another example of the UPS engaging a substrate in response to stress is provided by the RING-type E3 ligases RING domain Ligase 1 (RGLG1) and RGLG2, which regulate the abundance of ethylene response factor 53 (ERF53; Cheng et al., 2012). ERF53 is a drought and salt-responsive AP2/ERF transcription factor (Nakano et al., 2006; Cheng et al., 2012). Loss of both *RGLG1* and *RGLG2* gene function increase drought tolerance, which is consistent with a role for the E3 ligases in regulating ERF53 abundance (Cheng et al., 2012). RGLG1 and RGLG2 interact with and ubiquitinate ERF53 in *in vitro* assays (Cheng et al., 2012). In addition, overexpression of *ERF53* in *rglg1rglg2* plants further enhances drought tolerance of the double mutant and the transcription factor is stable in *rglg1rglg2* plants (Cheng et al., 2012). The RGLG proteins are suggested to be myristoylated and localized predominantly to the plasma membrane, while ERF53 is nuclear localized (Yin et al., 2007; Cheng et al., 2012). Although loss of the predicted myristoylation site disrupts RGLG2 membrane localization, the mutant E3 does not localize to the nucleus (Yin et al., 2007). Whether or not myristoylation regulates E3 ligase activity remains to be seen. However, salt stress does induce the translocation of RGLG2 to the nucleus where it interacts with ERF53 (Cheng et al., 2012). This suggests that RGLG2-mediated degradation of ERF53 occur in response to abiotic stress.

## NON-PROTEOLYTIC FUNCTIONS OF UBIQUITIN DURING ABIOTIC STRESS SIGNALING

While the requirement for ubiquitin-dependent protein degradation during response to abiotic stresses is firmly established, the involvement of other types of ubiquitin modification is not well understood. Of interest are the non-proteolytic functions of modifications such as monoubiquitination and lysine-63 linked polyubiquitination. The rice RING-type E3 ligase *Oryza sativa* heat and cold induced 1 (OsHCI1) is involved in tolerance of heat stress (Lim et al., 2013). OsHCI1 is capable of attaching a single ubiquitin molecule to a number of interacting proteins including OsbHLH065, a basic/helix-loop-helix (bHLH) transcription factor. Golgi-localized OsHCI1 translocates to the nucleus of cells exposed to heat shock and nuclear-localized OsbHLH065 in observed in the cytosol when co-expressed with OsHCI1. A role for OsbHLH065 in abiotic stress responses has not been reported. However, it is postulated that OsHCI1-mediated relocalization of nuclear proteins such as OsbHLH065 promotes heat stress tolerance. Monoubiquitination of the boron transporter BOR1 occurs in the presence of high concentrations of boron (Kasai et al., 2011). Boron is an essential nutrient for plant growth and development. Boron deficiency negatively affects yield, and high concentrations are toxic to plants. Plants utilize BOR1 for boron uptake under boron-limiting conditions and overexpression enhances tolerance of boron stress (Takano et al., 2002; Miwa et al., 2006). Boron-induced monoubiquitination of BOR1 is essential for vacuolar sorting and degradation of the transporter (Kasai et al., 2011). RGLG2 interacts with the E2 enzyme AtUBC35 (also referred to as AtUBC13) and both enzymes can facilitate the formation of lysine-63 linked polyubiquitin chains (Kraft et al., 2005; Yin et al., 2007; Wen et al., 2008). Lysine-63 linked chains have non-proteolytic functions such as endocytosis and protein activation (Chen and Sun, 2009). However, lysine-63 polyubiquitination can also serve as a signal for proteasomal degradation (Saeki et al., 2009). As discussed above, RGLG2’s role in abiotic stress response involves targeting the transcription factor ERF53 for proteasomal degradation (Cheng et al., 2012). Of interest is (1) the requirement for RGLG2 generated lysine-63 polyubiquitin chains during stress response and (2) whether RGLG2 modifies ERF53 with the attachment of a lysine-63 or lysine-48 linked polyubiquitin chain. Although the examples are few, the pervasiveness of the ubiquitin modification system suggests that the different types of ubiquitination may regulate aspects of plant responses to abiotic stresses.

## UBIQUITIN-DEPENDENT REGULATION OF STRESS HORMONE SIGNALING

**FIGURE 2:**
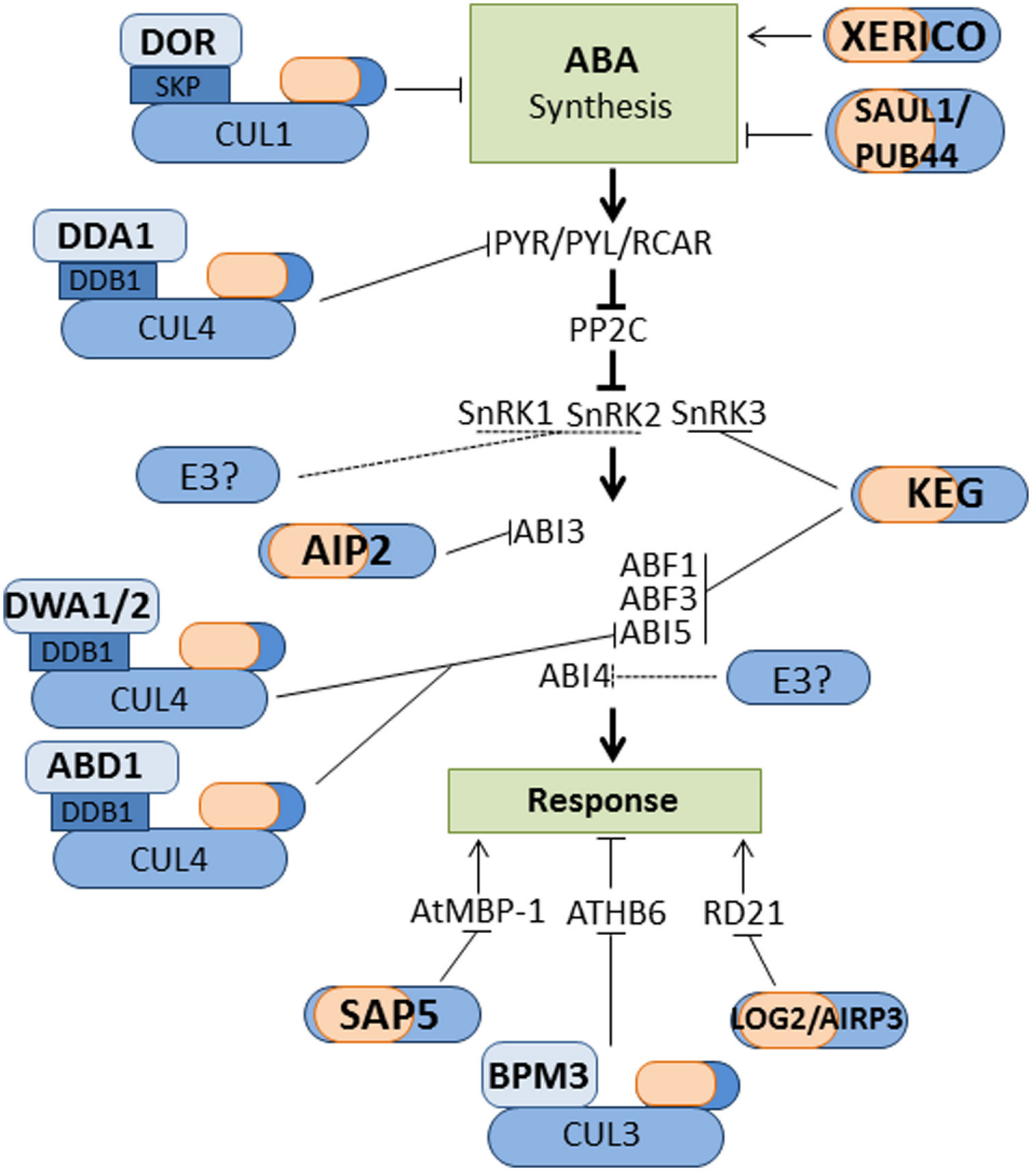
**Ubiquitin ligases that regulate ABA signaling.** Illustration of E3 ligases that regulate ABA synthesis, signal transduction and response. Not all E3 ligases are shown, mainly those with identified substrates. Question marks and dashed lines denote instances where proteasomal-dependent degradation is reported but the E3 ligase involved is unknown.

Plants utilize hormones to integrate endogenous and exogenous signals. A direct link has been demonstrated between the UPS and the production, perception, signal transduction, and outputs of these hormones. A surprising number of ubiquitin ligases have been shown to control the actions of stress hormones. For example, at least fourteen E3s have been linked to the regulation of ABA synthesis and signaling (**Figure 2**; Lee and Kim, 2011; Liu and Stone, 2011). Abiotic stresses such as drought and salinity increase cellular ABA levels via the induction of ABA biosynthetic genes including *9-cis-epoxycarotenoid dioxygenase 3* (*NCED3)* and *Arabidopsis aldehyde oxidase 3* (*AAO3*; Finkelstein, 2013). The U-box type E3 senescence-associated E3 ubiquitin ligase 1(SAUL1)/plant U-box (AtPUB) 44 negatively regulates ABA biosynthesis by targeting AAO3 for proteasome-dependent degradation (Raab et al., 2009; Salt et al., 2011). Drought tolerance repressor (DOR), a F-box protein that may participate in a CUL1 based RING E3 ligase, is a negative regulator of ABA-mediated responses (Zhang et al., 2008). Drought stressed *dor* plants exhibit enhanced expression of *NCED3* and increased cellular ABA levels. The stress-induced expression of *NCED3* is also enhanced by overexpression of the RING-type E3 XERICO, which is accompanied by increased ABA levels and improved tolerance of drought stress (Ko et al., 2006).

Perception of ABA is mediated by a suite of receptors named pyrabactin resistance 1(PYR1)/PYR1-like (PYL)/Regulatory component of ABA receptor (RCAR; Park et al., 2009; Santiago et al., 2009; **Figure 2**). ABA-bound PYR/PYL/RCAR receptors interact with and inhibit protein phosphatase type 2Cs (PP2Cs), which prohibits the dephosphorylation of sucrose non-fermenting1-related protein kinases (SnRKs; **Figure 2**; Fujii et al., 2009; Ma et al., 2009; Park et al., 2009). The ABA-activated SnRKs are then able to phosphorylate and activate transcription factors and other regulatory proteins involved in facilitating ABA-mediated process required for abiotic stress tolerance (Fujii et al., 2009; Rodrigues et al., 2013). Members of all three SnRK subfamilies, SnRK1, SnRK2, and SnRK3, have been implicated in mediating ABA response/signaling (Fujii et al., 2009; Lyzenga et al., 2013; Rodrigues et al., 2013). PYR/PYL/RCAR, PP2C, and SnRK proteins are considered the core components of the ABA signaling network (Weiner et al., 2010). As shown in **Figure 2**, the UPS regulate the abundance of many of these core components. A search for ubiquitinated proteins in *Arabidopsis* isolated ABA receptor PRY1 and SnRK kinases, SnRK1.1, SnRK2.4, and SnRK2.6/Open Stomata 1 (OST1; Kim et al., 2013). Ubiquitination of the identified targets increased after treatment with proteasome inhibitors, which suggests degradation by the 26S proteasome. De-etiolated 1(DET1)- and DDB1-associated protein 1 (DDA1), which functions as the substrate receptor for a CUL4 based E3 ligase, have been shown to regulate the abundance of ABA receptors PYL4, PYL8, and PYL9 (Irigoyen et al., 2014). ABA prohibits the DDA1-mediated degradation of PYL8 via reducing the ubiquitination of the receptor (Irigoyen et al., 2014). Calcineurin B-like Interacting protein kinase 26 (CIPK26), which belongs to the SnRK3 subfamily, is a positive regulator of ABA signaling (Lyzenga et al., 2013). CIPK26 interacts with two PP2Cs, abscisic acid insensitive (ABI) 1, and ABI2, phosphorylate the ABA-responsive transcription factor ABI5 *in vitro* and seedlings overexpressing CIPK26 are hypersensitive to ABA (Lyzenga et al., 2013). The RING-type E3 ligases, Keep on Going (KEG) interacts with CIPK26 targeting the kinase for degradation by the 26S proteasome. 

ABA-mediated responses, such as growth arrest of early seedlings exposed to stress conditions, involve the up or down-regulation of a large number of genes (Seki et al., 2002; Finkelstein, 2013). Changes in ABA-responsive gene expression are mediated by a number of transcription factors including members of the basic leucine zipper (bZIP), AP2/ERF, R2R3, and B3 families (Finkelstein, 2013). The UPS regulates ABA-responsive transcription by modulating the abundance of many of these transcription factors (**Figure 2**). The abundance of the nucleo-cytoplasmic bZIP transcription factor ABI5 is modulated by KEG (**Figure 2**). ABI5 promote the growth arrest of young seedlings exposed to stress conditions (Lopez-Molina et al., 2001). In the absence of stress, KEG is required to maintain low levels of ABI5 to ensure seedling establishment (Stone et al., 2006; Liu and Stone, 2010). KEG, a *trans*-Golgi network/cytosol-localized E3, ubiquitinates and targets ABI5 for degradation within the cytosol, which would prohibit accumulation of the transcription factor in the nucleus and activation of ABA responses (Gu and Innes, 2011; Liu and Stone, 2013). Elevated levels of ABA promote ABI5 accumulation via increased gene expression and decreased protein turnover. ABA-dependent stabilization of ABI5 protein involves KEG self-ubiquitination and proteasomal degradation (Liu and Stone, 2010). KEG also targets bZIP transcription factors ABRE-binding factors (ABF) 1 and ABF3 for degradation via the 26S proteasome (Chen et al., 2013). Similar to ABI5, ABA prohibits the proteasomal-dependent turnover of ABF1 and ABF3. Compared to other ABA mutants, the phenotype of *keg* seedlings is quite severe and growth arrest occurs in the absence of the hormone. The fact that KEG mediates the degradation of multiple components (CIPK26, ABI5, and ABF1/3) of the ABA signaling pathway helps to explain the lethality of the *KEG* mutation. The abundance of ABI4, an AP2/ERF transcription factor, is also regulated by the 26S proteasome, however the E3 involved is not yet identified (Finkelstein et al., 2011). The R2R3-type transcription factor MYB30 negatively regulates ABA signaling (Zheng et al., 2012). MYB30 is targeted for proteasomal degradation by the RING-type E3 MYB30-Interacting E3 Ligase 1 (MIEL1; Marino et al., 2013). MYB30 is multifunctional with additional roles in cell death and pathogen resistance (Marino et al., 2013). MEIL1-mediated degradation of MYB30 suppresses defense signaling in non-infected plants (Marino et al., 2013). Whether or not MIEL1-mediated degradation of MYB30 modulates ABA signaling is yet to be determined.

A monomeric RING-type E3 and two CRLs have been implicated in attenuating ABA signaling. ABI3, a B3 transcription factor, is targeted for proteasomal degradation by the RING-type E3 ABI3-interacting protein 2 (AIP2; Zhang et al., 2005). *aip2-1* accumulate high levels ABI3 compared to wild type and are hypersensitive to ABA. *AIP2* transcript abundance increases in response to ABA application and this correlates with a decrease in ABI3 levels. Thus, ABA promotes the turnover of ABI3, which would assist in suppressing hormone signaling. Nuclear-localized DWD hypersensitive to ABA 1 (DWA1), DWA2, and ABA-hypersensitive DCAF1 (ABD1) negatively regulates ABA signaling by promoting the turnover of ABI5 (Lee et al., 2010; Seo et al., 2014). DWA1, DWA2 and ABD1 proteins function as the substrate-recruiting component of CUL4 based RING E3 ligases (Lee et al., 2010; Seo et al., 2014). ABA treated *dwa1/dwa2* seedlings accumulate higher levels of ABI5 compared to wild type and the double mutants display hypersensitivity to ABA. ABI5 does not accumulate in *dwa1dwa2* in the absence of ABA, which is consistent with the CRL targeting the transcription factor for degradation in the presence of the hormone. Similarly, loss of *ABD1* results in hypersensitivity to ABA, and accumulation of ABI5 following exposure to the hormone (Seo et al., 2014). The BTB protein BMP3, which functions as the substrate-recruiting component of CUL3 based E3 ligase, regulates the abundance of AtHB6, a homeobox-leucine zipper transcription factor. ATHB6 is a negative regulator of ABA response (Himmelbach et al., 2002; Lechner et al., 2011). BMP3 promotes the proteasome-dependent degradation of ATHB6 under non-stress conditions (Lechner et al., 2011). ABA prohibits the turnover of ATHB6. The ABA-induced stabilization of ATHB6 may serve to attenuate ABA responses.

In addition to the above mentioned ubiquitin ligase, many other E3 ligases have been found to be involved in ABA responses. Substrate proteins have been identified for only few of these E3 ligases. Stress Associated Protein 5 (AtSAP5) is an A20/AN1-type zinc finger protein with E3 ligase activity (Kang et al., 2011). AtSAP5 mediate the proteasome-dependent degradation of *Arabidopsis* MBP-1-like protein (AtMBP-1), a positive regulator of ABA responses (Kang et al., 2013). The RING-type E3 ABA-Insensitive RING Protein 3(AtAIRP3)*/*Loss of GDU2 (LOG2) is a positive regulator of ABA-mediated stress responses (Kim and Kim, 2013). AtAIRP3*/*LOG2 interacts and ubiquitinates Glutamine Dumper1 (GDU1) and responsive to dehydration 21 (RD21). AtAIRP3/LOG2 ubiquitination of GDU1 is non-proteolytic and regulates the export of amino acids from plant cells (Pratelli et al., 2012). Whereas, AtAIRP3*/*LOG2 targets RD21 for degradation via the 26S proteasome (Kim and Kim, 2013). RD21 is drought-inducible Cys proteinase (Kim and Kim, 2013). However, it is not known if AtAIRP3/LOG2-mediated degradation of RD21 modulates drought tolerance or ABA responses. ABA-related E3 ligases with no known substrates include U-box-type E3s AtPUB9, AtPUB18, and AtPUB19. Down-regulation of *AtPUB9, AtPUB18*, and *AtPUB19* results in hypersensitivity to ABA, which suggests that the U-box-type E3s are negative regulators of ABA signaling (Samuel et al., 2008; Liu et al., 2011). Interestingly, in the presence of ABA, AtPUB9 is translocated from the nucleus to the plasma membrane. The significance of the ABA-induced relocalization is not known, however, the change in subcellular location may serve to inhibit E3 activity and promote ABA responses. The RING type E3s Salt and Drought Induced RING Finger 1(SDIR1), *Arabidopsis* ABA-insensitive RING protein 1(AtAIRP1), RING-H2 E3 ligase (RHA) 2a, and RHA2b are all positive regulators of ABA-mediated stress responses (Zhang et al., 2007; Bu et al., 2009; Ryu et al., 2010; Li et al., 2011). The identification of substrates for these orphan E3 ligases will shed further light on how the UPS facilities plant responses to and tolerance of adverse environmental conditions.

## Conflict of Interest Statement

The author declares that the research was conducted in the absence of any commercial or financial relationships that could be construed as a potential conflict of interest.
